# Lessons Learned From European Health Data Projects With Cancer Use Cases: Implementation of Health Standards and Internet of Things Semantic Interoperability

**DOI:** 10.2196/66273

**Published:** 2025-03-24

**Authors:** Amelie Gyrard, Somayeh Abedian, Philip Gribbon, George Manias, Rick van Nuland, Kurt Zatloukal, Irina Emilia Nicolae, Gabriel Danciu, Septimiu Nechifor, Luis Marti-Bonmati, Pedro Mallol, Stefano Dalmiani, Serge Autexier, Mario Jendrossek, Ioannis Avramidis, Eva Garcia Alvarez, Petr Holub, Ignacio Blanquer, Anna Boden, Rada Hussein

**Affiliations:** 1 Trialog Paris France; 2 Ludwig Boltzmann Institute for Digital Health and Prevention Salzburg Austria; 3 Discovery Research - ScreeningPort Fraunhofer Institute for Translational Medicine and Pharmacology Hamburg Germany; 4 Fraunhofer Cluster of Excellence for Immune-Mediated Diseases Frankfurt Germany; 5 Department of Digital Systems University of Piraeus Piraeus Greece; 6 Lygature Utrecht The Netherlands; 7 Diagnostic and Research Center for Molecular Biomedicine Diagnostic and Research Institute of Pathology Medical University of Graz Graz Austria; 8 Siemens Foundational Technologies Brasov Romania; 9 La Fe University Hospital Valencia Valencia Spain; 10 Monasterio Research Hospitals Pisa Italy; 11 Deutsches Forschungszentrum für Künstliche Intelligenz GmbH Bremen Germany; 12 Health Data Hub Paris France; 13 Ubitech Athens Greece; 14 Biobanking and Biomolecular Resources Research Infrastructure – European Research Infrastructure Consortium Graz Austria; 15 Universitat Politècnica de València València Spain; 16 Department of Clinical Pathology Centre for Medical Image Science and Visualization Linköping University Linköping Sweden

**Keywords:** artificial intelligence, cancer, European Health Data Space, health care standards, interoperability, AI, health data, cancer use cases, IoT, Internet of Things, primary data, diagnosis, prognosis, decision-making

## Abstract

The adoption of the European Health Data Space (EHDS) regulation has made integrating health data critical for both primary and secondary applications. Primary use cases include patient diagnosis, prognosis, and treatment, while secondary applications support research, innovation, and regulatory decision-making. Additionally, leveraging large datasets improves training quality for artificial intelligence (AI) models, particularly in cancer prevention, prediction, and treatment personalization. The European Union (EU) has recently funded multiple projects under Europe’s Beating Cancer Plan. However, these projects face challenges related to fragmentation and the lack of standardization in metadata, data storage, access, and processing. This paper examines interoperability standards used in six EU-funded cancer-related projects: IDERHA (Integration of Heterogeneous Data and Evidence Towards Regulatory and Health Technology Assessments Acceptance), EUCAIM (European Cancer Imaging Initiative), ASCAPE (Artificial Intelligence Supporting Cancer Patients Across Europe), iHelp, BigPicture, and the HealthData@EU pilot. These initiatives aim to enhance the analysis of heterogeneous health data while aligning with EHDS implementation, specifically for the EHDS for the secondary use of data (EHDS2). Between October 2023 and July 2024, we organized meetings and workshops among these projects to assess how they adopt health standards and apply Internet of Things (IoT) semantic interoperability. The discussions focused on interoperability standards for health data, knowledge graphs, the data quality framework, patient-generated health data, AI reasoning, federated approaches, security, and privacy. Based on our findings, we developed a template for designing the EHDS2 interoperability framework in alignment with the new European Interoperability Framework (EIF) and EHDS governance standards. This template maps EHDS2-recommended standards to the EIF model and principles, linking the proposed EHDS2 data quality framework to relevant International Organization for Standardization (ISO) standards. Using this template, we analyzed and compared how the recommended EHDS2 standards were implemented across the studied projects. During workshops, project teams shared insights on overcoming interoperability challenges and their innovative approaches to bridging gaps in standardization. With support from HSbooster.eu, we facilitated collaboration among these projects to exchange knowledge on standards, legal implementation, project sustainability, and harmonization with EHDS2. The findings from this work, including the created template and lessons learned, will be compiled into an interactive toolkit for the EHDS2 interoperability framework. This toolkit will help existing and future projects align with EHDS2 technical and legal requirements, serving as a foundation for a common EHDS2 interoperability framework. Additionally, standardization efforts include participation in the development of ISO/IEC 21823-3:2021—Semantic Interoperability for IoT Systems. Since no ISO standard currently exists for digital pathology and AI-based image analysis for medical diagnostics, the BigPicture project is contributing to ISO/PWI 24051-2, which focuses on digital pathology and AI-based, whole-slide image analysis. Integrating these efforts with ongoing ISO initiatives can enhance global standardization and facilitate widespread adoption across health care systems.

## Introduction

Cancer is one of the main causes of death in Europe and worldwide, after cardiovascular diseases. According to the World Health Organization (WHO), cancer is the second leading cause of death and morbidity in Europe, with more than 3.7 million new cases and 1.9 million deaths each year [1]. In response to the urgent need to renew European political commitment to tackle cancer, Europe’s Beating Cancer Plan (EBCP; 2021-2027) was launched and structured around 4 key action areas: prevention, early detection, diagnosis and treatment, and the quality of life of patients with cancer and cancer survivors [2]. Since 2021, the European Commission (EC) has supported collaborative projects focused on cancer diagnostics and treatment using high-performance computing and artificial intelligence (AI). To maximize the potential of data and digitalization, the EBCP also addressed the interactions and alignment of cancer data projects and initiatives with the European Health Data Space (EHDS). In autumn 2023, the EC organized 3 online workshops on the reuse of health data resources in the field of cancer research and recently published the results of these workshops [3], where “fragmentation and the lack of standardization in metadata, data storage, access, and processing” were identified as key challenges facing data-driven cancer projects. The nascent infrastructure for the application of AI in medical imaging (European Cancer Imaging Initiative [EUCAIM]) reported on the experiences of 5 projects developing big data infrastructures that will enable European, ethical, General Data Protection Regulation (GDPR)–compliant, quality-controlled, and cancer-related medical imaging platforms, where both large-scale data and AI algorithms will coexist [4]. These projects include the following:

1. Chameleon: a project focused on developing AI algorithms for cancer diagnosis and prognosis.
2. EuCanImage: a project aimed at creating a large-scale cancer image database.
3. ProCAncer-I: a project focused on developing AI-based tools for personalized cancer treatment.
4. Incisive: a project contributing a significant amount of cancer image data to EUCAIM.
5. Primage: a project focused on developing AI-based image analysis techniques for cancer diagnosis.

These projects along with the RadioVal project established the AI for Health Imaging (AI4HI) network to develop cancer imaging data repositories and AI solutions based on medical imaging to improve clinical practice.

Table 1 summarizes the list of projects that participated in the EC workshop entitled “Landscaping data-driven projects and initiatives in the cancer field–rationale and directions for better collaboration and integration” [3], as well as the established project-level synergies and collaborations among ongoing projects.

The workshop also addressed the current challenges facing data-driven cancer projects [3], gaps existing in existing standards, and recommendations for future semantic interoperability (given in Table 2).

**Table 1:** Collaborations and synergies among the European Union’s existing cancer projects.

Collaboration scope	Synergy projects
Data representation and interoperability	HealthData@EU pilot and CanSERV
Infrastructure and services for benchmarking	EOSC4Cancer, EUCAIM^a^, and TEF-Health
Federated data infrastructure and data structure	EUCAIM, EOSC4Cancer, and GDI
Secure processing environment	SOLACE, EUCAIM, CanScreen-ECIS, IDERHA^b^, and Optima

^a^EUCAIM: European Cancer Imaging Initiative.

^b^IDERHA: Integration of Heterogeneous Data and Evidence Towards Regulatory and Health Technology Assessments Acceptance.

**Table 2:** Main challenges facing European Union cancer projects and recommendations.

Challenges [3]	Gaps in existing standards	Recommendations
Fragmentation and lack of standardization in metadata, data storage, access, and processing.	The DCAT-AP^a^ health extension is still under development by the HealthData@EU pilot.	TEHDAS^b^ JA^c^ recommendations to enhance interoperability within HealthData@EU—a framework for semantic, technical, and organizational interoperability.
Lack of access to diverse and high-quality datasets.	Data quality framework for primary care data sources is not sufficiently addressed in the EHDS^d^ framework.	Data quality frameworks provided by TEHDAS, EMA^e^, and QUANTUM projects.
Evolving legal landscape	Relevant standards to the AI^f^ Act are under development.	Participating in developing or extending the relevant standards.

^a^DCAT-AP: DCAT Application Profile for Data Portals in Europe.

^b^TEHDAS: Towards the European Health Data Space.

^c^JA: joint action.

^d^EHDS: European Health Data Space.

^e^EMA: European Medicines Agency.

^f^AI: artificial intelligence.

## HSbooster Health Project Cluster in Cancer

In this work, we focus on the interoperability challenges and existing gaps in health care standards building on the challenges and synergies highlighted in the EC workshop [3]. Through the EC European Standardization Booster (HSbooster.eu) initiative, we create synergy among six cancer data-driven projects (ie, IDERHA [Integration of Heterogeneous Data and Evidence towards Regulatory and Health Technology Assessments Acceptance], EUCAIM, ASCAPE [Artificial Intelligence Supporting Cancer Patients Across Europe], iHelp, BigPicture, and the HealthData@EU pilot project) by using health standards. These 6 innovative projects aim to transform digital health in oncology by leveraging advanced technologies and collaborative frameworks for enhancing cancer diagnosis, treatment, and research, improving patient outcomes, and accelerating scientific advancements. We initially created a template for the EHDS for the secondary use of data (EHDS2) interoperability framework based on the new European Interoperability Framework (EIF). The recommended standards and governance model from the joint action (JA) Towards the European Health Data Space (TEHDAS) [5] for the secondary use of data were then used to harmonize the standards in the template (given in Textbox 1).

Starting October 2023, we conducted several meetings and workshops among the 6 projects to elucidate how they adopt health standards and Internet of Things (IoT) semantic interoperability. This included examining interoperability standards for health data, knowledge graphs–related technologies, the Smart Applications Reference Ontology (SAREF), the data quality framework (DQF), patient-generated health data (PGHD), AI reasoning, federated approaches, security, and privacy.

As per the TEHDAS recommendations, we compared the health-standardized models, ontologies, and terminologies used in these projects, including, Health Level 7 (HL7) Fast Healthcare Interoperability Resources (FHIR), Open Health Data Science and Informatics (OHDSI), Observational Medical Outcomes Partnership (OMOP)–Common Data Model (CDM), Digital Imaging and Communications in Medicine (DICOM), International Organization for Standardization (ISO) Technical Committee (TC) 215, and World Wide Web Consortium (W3C) Data Catalog Vocabulary (DCAT).

As EHDS is still evolving and its implementation is still under development, this study aims to examine how the 6 projects implement the recommended EHDS2 standards. Consequently, we introduce a new template to support the EHDS2 interoperability framework and highlight the results of comparing data standards across projects. By summarizing the lessons learned, we provide recommendations for future directions in EHDS2 implementation.

**European Health Data Space for European Interoperability Framework.**

**European Health Data Space**

- The European Health Data Space (EHDS) [6] aims to harmonize health data usage across Europe [7]. It includes comprehensive rules, standards, practices, infrastructures, and a robust governance framework to improve health care delivery, drive research and innovation, and inform policymaking.
- A central focus of the EHDS is empowering patients by providing them greater digital access and control over their health data, fostering a more transparent and efficient health care system. In addition, the EHDS seeks to standardize health data across member states, ensuring interoperability, enhancing clinical outcomes, and accelerating medical research and innovation through a unified dataset.
- The governance framework ensures data privacy and security, addresses ethical concerns, and fosters stakeholder trust. The EHDS also aims to streamline regulatory processes and support cross-border health care initiatives by aligning national and regional policies.
- Therefore, the EHDS will significantly impact Europe’s digital health landscape by promoting a more connected, efficient, and patient-centered health care ecosystem.

**Joint Action Toward the European Health Data Space**

- The Toward the European Health Data Space (TEHDAS) project [5] is a joint action that developed European principles for the secondary use of health data, involving 25 countries. It advocates for using existing International Organization for Standardization (ISO) standards to ensure consistency and interoperability. TEHDAS focuses on data interoperability, identifying standards for data discovery and common data models. A synthesis table details essential standards including DCAT Application Profile for Data Portals in Europe (DCAT-AP) [8], INSPIRE, FairSharing, Systematized Nomenclature of Medicine Clinical Terms (SNOMED CT), and Health Level 7 (HL7) Fast Healthcare Interoperability Resources (FHIR).
- The project addresses syntactic interoperability, providing guidelines on data structure and format for seamless sharing across systems. In addition, TEHDAS emphasizes data quality, proposing frameworks to regulate and enhance the reliability of health data for secondary uses, such as research and policymaking. Overall, TEHDAS’s work supports a standardized and interoperable EHDS, leading to improved health care, research, and innovation in Europe.

**European Interoperability Framework**

- The new European Interoperability Framework (EIF) [9] adopted on March 23, 2017, fosters electronic communication and information exchange among European public administrations by providing common models, principles, and recommendations. The EIF delineates four layers of interoperability challenges: legal, organizational, semantic, and technical. This paper concentrates on the semantic interoperability challenge, encompassing both semantic and syntactic aspects. Semantic interoperability ensures that the meaning of exchanged data is preserved and understood across different systems. This can be achieved through the use of shared vocabularies and schemas. On the other hand, syntactic interoperability involves defining the grammar and format for data exchange.
- The EIF provides guidance for European public administrations to improve interoperability, ensuring smooth data exchange and efficient digital service delivery. Conversely, ISO 23903 concentrates on semantic interoperability within Information and Communication Technology (ICT) systems, aiming to unify the interpretation of information across various platforms.
- Aligning the EIF with ISO 23903 synchronizes European interoperability principles with semantic standards, fostering easier communication and cooperation among public administrations. This alignment ensures both technical harmony and uniformity in the meaning and semantics of exchanged data, thereby enhancing the efficiency and efficacy of digital service provision for citizens and businesses across Europe.

## Template for the EHDS2 Interoperability Framework Based on the EIF

**Figure 1:**
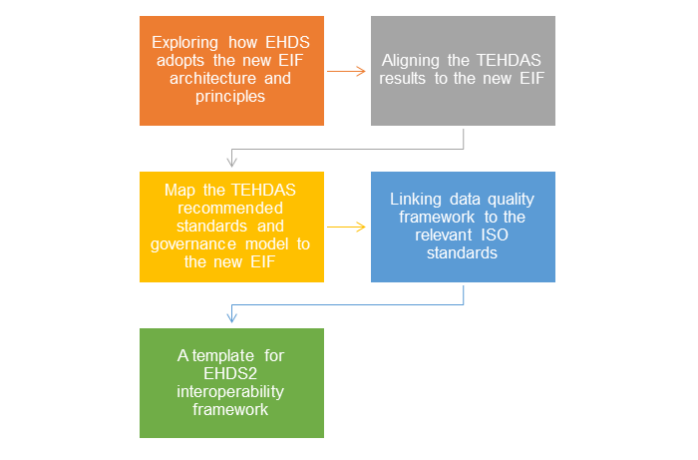
The process for creating the proposed template for the EHDS2 interoperability framework. EHDS: European Health Data Space; EHDS2: European Health Data Space for the secondary use of data; EIF: European Interoperability Framework; ISO: International Organization for Standardization; TEHDAS: Towards the European Health Data Space.

Figure 1 shows the process of creating the template for the EHDS2 interoperability framework.

### Exploring How EHDS Adopts the New EIF Architecture and Principles

Recent studies showed the importance of adopting the EIF in establishing the EHDS interoperability framework [7,10,11] (as shown in Textbox 1). In addition, several studies have investigated the interoperability requirements for heterogeneous health information systems, as well as the associated health care standards [12,13].

### Aligning the TEHDAS Results to the New EIF

The TEHDAS JA involves 25 European countries in developing the principles that will shape EHDS2 by providing guidance and recommendations on interoperability, data quality, and standards (as shown in panel 2 in Textbox 1). In 2023, the TEHDAS JA published a report titled “Options for governance models for the European Health Data,” which discusses EHDS governance using the EIF [14]. In addition, they assessed 19 standards that provide a layer of semantic interoperability, supporting the cataloging of data sources and the exchange of data between different nodes [15].

### Mapping the TEHDAS Recommended Standards and Governance Model to the New EIF

**Figure 2:**
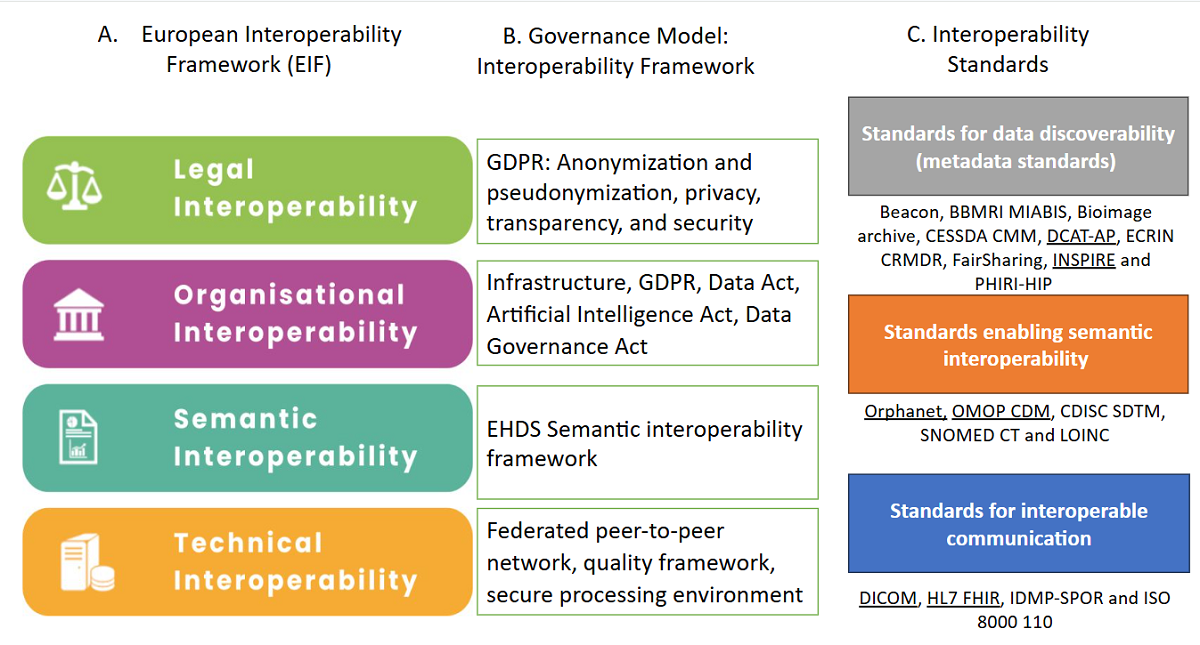
Template for the EHDS2 interoperability framework based on the TEHDAS results: (A) EIF layers; (B) TEHDAS governance model and associated interoperability framework [14]; and (C) TEHDAS 19 standards evaluation using the EIF, with the selected standards that “passed the EIF criteria evaluation” underlined [15]. DCAT-AP: DCAT Application Profile for Data Portals in Europe; DICOM: Digital Imaging and Communications in Medicine; EHDS: European Health Data Space; EHDS2: European Health Data Space for the secondary use of data; EIF: European Interoperability Framework; FHIR: Fast Health Interoperability Resources; GDPR: General Data Protection Regulation; HL7: Health Level 7; ISO: International Organization for Standardization; LOINC: Logical Observation Identifiers Names and Codes; OMOP-CDM: Observational Medical Outcomes Partnership Common Data Model; SNOMED CT: Systematized Nomenclature of Medicine–Clinical Terminology.

Following the recommendation of the EC workshops on landscaping data-driven projects and initiatives in cancer [3], we adopted the TEHDAS results on recommended standards for EHDS2 interoperability, as well as the principles of data quality frameworks. We then mapped these results to the new EIF interoperability framework and linked the data quality framework to the corresponding ISO standards. Figure 2 shows how the TEHDAS results are mapped to the new EIF interoperability framework. Notably, TEHDAS framed the recommended EHDS2 interoperability into 3 categories of standardization:

1. Data discoverability (DCAT-AP)
2. Enabling semantics interoperability (OMOP-CDM)
3. Health data exchange (DICOM for imaging data and FHIR for health records).

The template shows the importance of incorporating and harmonizing the underpinning standards to comply with new regulations, such as the AI Act [16]. As a result, integrating advanced AI methodologies within the proposed framework enhances TEHDAS principles and advances beyond EIF by embedding robust data quality frameworks, enabling federated learning for secure and scalable data sharing, and incorporating privacy-preserving mechanisms that comply with GDPR. This framework delivers actionable templates that bridge theoretical interoperability principles with real-world AI applications, such as cancer diagnostics and personalized treatment. By aligning technical standards with semantic requirements and supporting adaptability to evolving regulations, such as the European AI Act, the framework provides a scalable, future-ready solution for health care interoperability and AI-driven innovation.

The EIF does not classify standards according to interoperability layers (technical, semantic, legal, and organizational). However, we need to consider the relevant standards for data privacy and quality assessment [10].

Furthermore, the work is a foundation for creating an interactive tool for the EHDS2 Interoperability Framework, which has been submitted to JMIR as part 2 (Towards EHDS2 interoperability framework: An interactive EIF-based standards compliance toolkit for AI-driven projects).

### Linking DQF to the Relevant ISO Standards

In May 2024, the Big Data Value Association (BDVA) published a study entitled “Elevating Data Quality A Paradigm Shift for Data Spaces and AI Needs” to explore the relationships between data quality and data spaces with respect to the AI Act [17]. The study proposed that the data quality and utility label for EHDS should comply with data documentation, technical quality, data quality management processes, coverage, access and provision, and data enrichment procedures.

**Figure 3:**
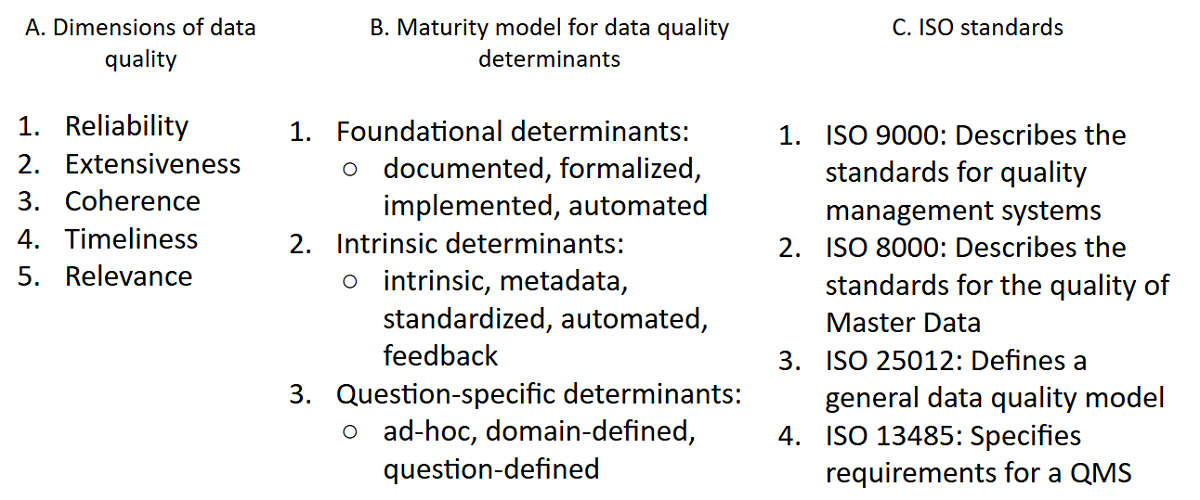
European Medicines Agency data quality framework in terms of the (A) dimensions of data quality, (B) maturity levels, and (C) related ISO standards. ISO: International Organization for Standardization; QMS: quality management system.

From the EHDS2 perspective, the TEHDAS JA provided a generic DQF [18], which includes both technical quality elements and six utility dimensions: relevance, accuracy and reliability, coherence, coverage, completeness, and timelines. This was followed by the publication of associated recommendations affecting data quality and utility implementation in HealthData@EU [19]. Based on these deliverables, the European Medicine Agency (EMA) published its own DQF for EU medicines regulation [20]. This publication provides an analysis of the data quality actions and metrics, as well as a maturity model, to guide the evolution of automation to support data-driven regulatory decision-making (as shown in Figure 3) [20].

Ensuring the accuracy, completeness, consistency, and reliability of cancer research data is of utmost importance, and adherence to data quality standards plays a crucial role in achieving this goal. These standards not only help maintain data integrity but also promote data interoperability through the use of standardized data models and vocabularies. This enables seamless data exchange and integration across different projects and platforms. In addition, adhering to these standards supports informed decision-making by providing high-quality data for clinical decisions, research insights, and policymaking related to cancer treatment and prevention. Compliance with international legal and regulatory requirements is also ensured, upholding proper data governance and ethical data usage. Finally, establishing a unified framework for data quality encourages collaboration among various stakeholders within the cancer research community. We compare data quality standards in Table S4 in Multimedia Appendix 1.

## Landscape of the Involved Projects and Used Standards

In this study, we used the EHDS2 interoperability framework template to compare 6 projects with cancer use cases where AI is applied (as shown in panel 4 in Textbox 2; Table 3). The selected projects vary in scope, cancer domain, categories of health data, scale of infrastructure, AI implementation approach, and time spans [21].

We focused on semantic interoperability standards (as shown in Textbox 3), recommended by the TEHDAS JA and related ISO standards. The analysis explored how health standards support health ontologies, for example, how HL7 FHIR supports the Findability, Accessibility, Interoperability, and Reusability (FAIR) principles through the FHIR for FAIR implementation guide [28].

**Introduction to six key projects advancing oncology through digital health.**

We explore 6 innovative projects that are transforming digital health in oncology. By leveraging advanced technologies and collaborative frameworks, these projects aim to enhance cancer diagnosis, treatment, and research, improving patient outcomes, and accelerating scientific advancements:

- IDERHA (Integration of Heterogeneous Data and Evidence Towards Regulatory and Health Technology Assessments Acceptance) [22] (2023-2028): IDERHA advances digital health in the context of lung cancer. It seeks to enhance health care delivery and improve patient outcomes by integrating various health data sources to support effective clinical decision-making and the development of improved treatments.
- BigPicture [23] (2021-2027): BigPicture aims to revolutionize pathology practice by establishing a repository of high-quality, annotated images, and developing artificial intelligence (AI) tools to improve diagnostic accuracy and efficiency. Data are collected from all types of tissues, including 1 million clinical samples and 2 million nonclinical samples from toxicology studies.
- EUCAIM (European Cancer Imaging Initiative) [24] (2023-2026): EUCAIM provides a federated infrastructure for sharing and analyzing cancer images. This supports AI research and clinical practices to advance cancer diagnosis and treatment.
- iHelp [25] (2021-2024): The iHelp project is dedicated to developing an intelligent, AI-driven health monitoring platform for early disease detection and management. It offers personalized health care solutions that enhance patient engagement and outcomes, with a specific focus on pancreatic cancer.
- ASCAPE (Artificial Intelligence Supporting Cancer Patients Across Europe) [26] (2020-2023): ASCAPE leverages AI to enhance the quality of life for patients with cancer. By using advanced analytics on health data, the project develops predictive models and personalized treatment plans to support patient care and survivorship.
- HealthData@EU Pilot [27] (2022-2024): This project aims to establish a technical infrastructure to enable cross-border health data access and use within the European Union, empowering the secondary use of health data more broadly. This initiative supports the creation of a cohesive digital health ecosystem, fostering innovation and improving health care services across Europe.

**Table 3:** Six projects with cancer use cases applying artificial intelligence.

Project	Duration	Cancer domain	AI^a^ or ML^b^	Comments
IDERHA^c^	2023-2028	Lung cancer	Federated machine learning	Under implementation
BigPicture	2021-2027	Pan-cancer whole slide images	Central repository for digital pathology and platform for AI development	Ongoing
EUCAIM^d^	2023-2026	Pan-cancer images	Federated Research Infrastructure	Ongoing
iHelp	2021-2024	Pancreatic cancer	Explainable AI, deep neural networks, predictive algorithms, ML techniques, and federated queries on distributed infrastructures	Ended on June 30, 2024
ASCAPE^e^	2020-2023	Breast and Prostate cancer	Explainable AI, federated deep learning, and ML on homomorphically encrypted data	Finished
HealthData@EU pilot	2022-2024	Colorectal cancer and other nonrelated cancer use cases	Federated query in noncancer use case: machine learning for analysis of care pathways	Pilot for EHDS2

^a^AI: artificial intelligence.

^b^ML: machine learning.

^c^IDERHA: Integration of Heterogeneous Data and Evidence Towards Regulatory and Health Technology Assessments Acceptance.

^d^EUCAIM: European Cancer Imaging Initiative.

^e^ASCAPE: Artificial Intelligence Supporting Cancer Patients Across Europe.

**Advancing semantic interoperability in digital health: a landscape of standards used in the involved projects.**

Our attention is drawn to the critical role that standards play in examining semantic interoperability within the digital health realm. These standards form the foundation for seamless data exchange, ensuring consistency and coherence across various health care platforms. The main standards are as follows:

- Health Level 7 (HL7) Fast Healthcare Interoperability Resources (FHIR) [29]: Positioned as a cornerstone in health care interoperability, FHIR stands out for its efficiency, adaptable architecture, and robust data exchange mechanisms, which facilitate smooth communication across disparate systems.
- DICOM (Digital Imaging and Communications in Medicine) [30]: DICOM is widely adopted in clinical and research settings, underscoring its effectiveness in enabling image exchange.
- Common Data Model (CDM)–OMOP (Observational Medical Outcomes Partnership) [31]: While not strictly a standard, CDM-OMOP allows for data harmonization and analysis. Its synergy with established standards enhances overall interoperability.
- International Organization for Standardization (ISO) [32]:
  - ISO TC 215 and CEN TC 251 Health Informatics: These cover a range of ISO standards, such as 13606 (5 parts), 27269, and 29585, alongside the Health Informatics Service Architecture (HISA)–compliant data models.
  - ISO 13606: This focuses on exchanging information on electronic health records between different health care information systems. It has a comprehensive structure for organizing clinical data, including patient demographics, medical history, diagnoses, treatments, and other relevant information.
  - ISO/AWI TR 24305: This guideline, derived from ISO standards 13940 and 13606, offers a structured HL7 FHIR implementation framework. It strengthens interoperability infrastructure, aligns with ISO norms, and promotes uniformity and adherence to best practices.
  - ISO 23903: Titled “Health Informatics - Interoperability and Integration Reference Architecture (IIRA),” this standard addresses the integration and interoperability challenges in health information systems and services, ensuring they can communicate effectively and share data seamlessly.
  - ISO 13972: Titled “Health Informatics - Detailed Clinical Models (DCM),” this standard focuses on the standardization and interoperability of clinical information models. DCMs are specifications that represent the clinical concepts and data elements used in electronic health records (EHRs) and health information systems.
- Internet of Things and eHealth coding standards
  - ETSI SmartM2M Smart Applications Reference Ontology (SAREF) [33]: Tailored for the eHealth and healthy aging domains, representing a specialization within standardization. Its integration expands interoperability across different targeted demographic groups.
  - Systematized Nomenclature of Medicine (SNOMED) [34]: SNOMED is a comprehensive clinical terminology system that captures, encodes, and shares EHRs and other health care data. It provides a standardized vocabulary for describing clinical concepts, enabling interoperability and semantic consistency across health care systems. SNOMED facilitates accurate and meaningful exchange of clinical information, supporting clinical decision-making, research, and public health reporting.
  - Logical Observation Identifiers Names and Codes (LOINC) [35]: LOINC is a standardized system for identifying and exchanging clinical laboratory test results and other clinical observations. It provides a universal set of codes and terms for describing laboratory tests, measurements, and observations, ensuring interoperability and semantic consistency in healthcare data exchange. LOINC enables seamless integration of laboratory data into EHRs and other health information systems.
  - International Classification of Diseases (ICD) [36]: It is promoted by the World Health Organization (WHO). ICD codes are used to define diagnoses for clinical treatment, medical billing, and statistics collections. It contains codes for diseases, signs and symptoms, abnormal findings, complaints, social circumstances, and external causes of injury or diseases. These codes help physicians in determining which conditions are relevant for clinical decisions, injuries, and causes of death. However, ICD has some limitations (eg, diabetes has more than 2 dozen different codes). Patient data recorded with ICD can be used for administrative functions, epidemiologic studies, research subject recruitment, interventional protocols, and clinical decision support systems.

The other standards regarding data quality, security, artificial intelligence (AI), and wearables are listed in the Multimedia Appendix 1.

**Abbreviations of semantic metadata or terminologies used by standards**

- ATC: Anatomical Therapeutic Chemical Classification System; Birnlex: Biomedical Research Integrated Domain Group; CPT: Current Procedural Terminology; ICD: International Classification of Diseases; ICDO: International Classification of Diseases for Oncology; NAACR: North American Association of Central Cancer Registries; NCIT: National Cancer Institute Thesaurus; OSIRIS: Open Source Infrared Imaging System; RADLEX: Radiology Lexicon; RDFS: Resource Description Framework Schema; SPARQL: SPARQL Protocol and RDF Query Language; RxNorm: Prescription Normative Terminology; UCUM: Unified Code for Units of Measure; UMLS: Unified Medical Language System.

## Comparing the Health Data Projects

To identify synergies among the 6 projects, we used the EHDS2 interoperability framework template to analyze adopted standards, highlighting similarities and differences in the implementation approach (detailed results given in Tables S1-S7 in Multimedia Appendix 1).

## Mapping the Projects to the Created Template for the EHDS2 Interoperability Framework

The EHDS2 interoperability framework template identified key standards and technologies for the implementation of EHDS2, including HL7 FHIR; DICOM; OMOP-CDM; upcoming standards developed by ISO TC 215, ISO TC212, CEN TC 251; and ontology technologies, including W3C DCAT-AP. The key standards used in 6 projects, their focus areas, and planned future implementations are summarized in Table 4.

**Table 4:** Overview of the used, innovated, and planned health data standards in the projects (using the template of the European Health Data Space for the secondary use of data [EHDS2] interoperability framework).

Project	Used standards	Planned or future standards
IDERHA	HL7^a^ FHIR^b^, DICOM^c^, and OMOP	ISO^d^ TC^e^ 215, DCAT-AP^f^: HealthDCAT-AP
BigPicture	DICOM, SNOMED^g^, and *ICD*	ISO TC 212 (digital pathology and AI^h^), and Kidney Biopsy Codes
EUCAIM^i^	Using its own hyperontology and common data model based on: DICOM, DICOM Seg, OMOP, FHIR, mCODE, DCAT-AP-Health, OSIRIS Ontologies used so far: LOINC^j^, SNOMED, UCUM^k^, RADLEX^l^, ICDO3, *ICD-10*^m^, CPT4, ICD10PCS, ATC^n^, NCIT^o^, Birnlex^p^, NAACR^q^, Cancer Modifier	—^r^
iHelp	HL7 FHIR, OMOP-CDM, ISO 27799:2016, SNOMED, LOINC, *ICD-9*, ICD-10, UMLS, SPARQL, RDFS^s^, RxNorm	—
ASCAPE	HL7 FHIR, ISO/CEN 13606, LOINC, SNOMED	—
HealthData@EU pilot	Observing and collecting standardization efforts rather than direct implementation	The first version of the Health DCAT-AP metadata standard is being developed in the project

^a^HL7: Health Level 7.

^b^FHIR: Fast Health Interoperability Resources.

^c^DICOM: Digital Imaging and Communications in Medicine.

^d^ISO: International Organization for Standardization.

^e^TC: Technical Committee.

^f^DCAT-AP**:** DCAT Application Profile for Data Portals in Europe.

^g^SNOMED: Systematized Medical Nomenclature for Medicine.

^h^AI: artificial intelligence.

^i^EUCAIM: European Cancer Imaging Initiative.

^j^LOINC: Logical Observation Identifiers Names and Codes.

^k^UCUM: Unified Code for Units of Measure.

^l^RADLEX: Radiology Lexicon.

^m^ICD-10: International Classification of Diseases, Tenth Revision, Clinical Modification.

^n^ATC: Anatomical Therapeutic Chemical classification system.

^o^NCIT: National Cancer Institute Thesaurus.

^p^Birnlex: Biomedical Research Integrated Domain Group.

^q^NAACR: North American Association of Central Cancer Registries.

^r^Not applicable.

^s^RDFS: Resource Description Framework Schema.

## Main Findings and Lessons Learned

All projects support the recommended standards identified by the TEHDAS JA for EHDS2 interoperability. Although the DICOM standard is widely used for collecting, storing, and transferring medical imaging data, it lacks important information required to identify relevant images because DICOM metadata are not standardized [37]. To overcome this challenge, the EUCAIM project CDM is built upon the FHIR resources ImagingStudy and ImagingSeries, the Medical Imaging–CDM extension of the OMOP-CDM, the ProCAncer-I imaging extension [38], and the OSIRIS imaging component. In this way, proper integration of imaging and clinical data was provided in alignment with the Integrating the Healthcare Enterprise [39].

The BigPicture project selected the DICOM format as a standard for archiving whole-slide images (WSI) to support back-and-forth conversion of proprietary file formats. The BigPicture profile for DICOM WSI is based on work by the DICOM pathology working group WG26. The format is designed to allow efficient storage of a large number of annotations, for example, generated by AI algorithms [40]. The project developed a Python tool (Wsidicom) to serve as a reference implementation of a DICOM WSI reader [41]. In addition, two tools, Opentile and Wsidicomizer, were developed to read other WSI formats and convert them to DICOM resulting in faster format conversion and maintained image quality [42].

To ensure structured data exchange and harmonized-consistent metadata interaction for data not captured in DICOM, BigPicture has developed additional metadata standards that define and comprise a set of substandards: (1) the data model (Common Mandatory Metadata Structure[CMMS]) comprising of all metadata, (2) a set of mandatory information that must be provided in relation to CMMS entities and their relations to each other, (3) a metadata file format that is used within BigPicture (flexible metadata file exchange format), and (4) a standard file structure of datasets containing metadata and data files that can be found on the repository. Standards are based on the European Genome-phenome Archive (EGA), and where possible existing standards have been incorporated (SNOMED, ICD for clinical data, Standardization for Exchange of Nonclinical Data [SEND] terminology, and International Harmonization of Nomenclature and Diagnostic Criteria (INHAND) nomenclature for nonclinical data)*.* Results from BigPicture, such as metadata formats or quality control criteria, will feed into the ISO/AWI 24051-2 “Medical laboratories—Part 2: Digital pathology and artificial intelligence (AI)-based image analysis.”

Regarding the metadata standards, the HealthData@EU pilot published a landscape analysis of available metadata catalogs and metadata standards [43]. The pilot also develops an extension of DCAT-AP: HealthDCAT-AP that will be adopted by EHDS2 projects, like IDERHA [44]. This standard could be adopted later as the mandatory metadata standard foreseen under the EHDS regulation. The EUCAIM project designed its Hyper Ontology [45] using FHIR, UMLS, SNOMED-CT, and OMOP-CDM vocabularies.

While data from wearables are available in high volumes, a substantial amount of data is lost because the usability of the data is governed and limited by proprietary data formats from an increasing number of manufacturers, which shows the necessity of standardization to enable interoperability [46]. Among the six projects and initiatives, IDERHA, iHelp, and ASCAPE use sensors and wearables technologies. The iHelp project uses the holistic health records (HHR) model to enable the aggregation of data from different sources, sensors, and online platforms to support the seamless integration of multiple health dimensions [47].

The HL7 FHIR standard-compatible data structures were used in the context of the iHelp project to integrate primary and secondary data and to compile them into the holistic health records FHIR model [47]. FHIR can be used for clinical data and also for streaming data from sensors [48]. The ASCAPE project successfully integrated EN/ISO 13606–standardized extracts from a patient mobile app into an electronic health record [49]. IDERHA plans to extend the OMOP-CDM to address the PGHD, including patient-reported outcomes and patient-reported experience measures.

**Figure 4:**
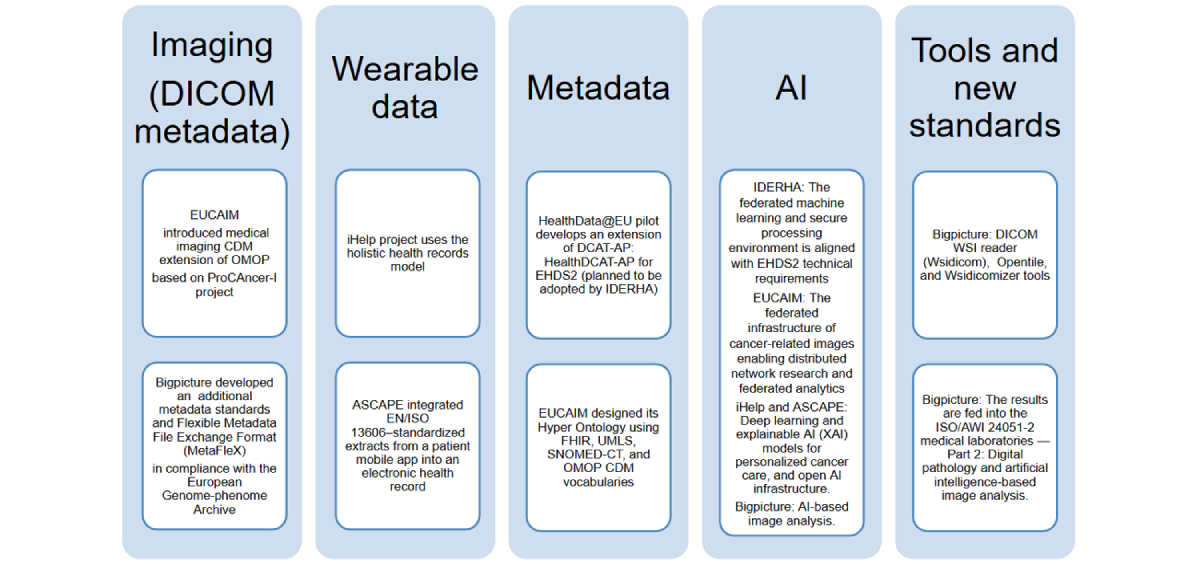
Key outputs and lessons learned from the six projects. AI: artificial intelligence; ASCAPE: Artificial Intelligence Supporting Cancer Patients Across Europe; AWI: approved work item; CDM: Common Data Model; DCAT-AP: DCAT Application Profile for Data Portals in Europe; DICOM: Digital Imaging and Communications in Medicine; EHDS: European Health Data Space; EHDS2: European Health Data Space for the secondary use of data; EUCAIM: European Cancer Imaging Initiative; HHR: holistic health record; IDERHA: Integration of heterogeneous Data and Evidence Towards Regulatory and Health Technology Assessments Acceptance; iHelp: Personalised Health Monitoring and Decision Support Based on Artificial Intelligence and Holistic Health Records; OMOP: Observational Medical Outcomes Partnership; SNOMED-CT: Systematized Nomenclature of Medicine Clinical Terms; UMLS: Unified Medical Language System; WSI: whole-slide imaging; ΧΑΙ: explainable artificial intelligence.

The BigPicture project highlighted that a dedicated standardization project focused on digital pathology is currently missing. It addressed the need for ISO/AWI 24051-2 “Medical laboratories—Part 2: Digital pathology and artificial intelligence (AI)-based image analysis” (under development, April 2024) that will build on ISO 20166-4:2021 “Molecular in vitro diagnostic examinations Specifications for pre-examination processes for formalin-fixed and paraffin-embedded (FFPE) tissue Part 4: In situ detection techniques” [50]. Figure 4 summarizes the main findings of the projects and how they can feed each other.

It is interesting to notice that the ETSI SmartM2M SAREF extensions for health and aging well (SAREF4EHAW) and wearables (SAREF4WEAR) are not known and not used on those six projects that we compared, even projects using IoT technologies with sensors, devices.

Key reasons for this gap include insufficient promotion, perceived relevance, integration challenges, and the presence of competitive standards. ETSI should enhance outreach, showcase successful implementations, collaborate with key projects, and simplify integration processes to improve adoption.

The other aspects, AI and federated learning, security and privacy, and data quality are covered by several approaches and standards. The adoption of federated standards and AI in cancer research projects across Europe represents a shift toward collaborative yet privacy-preserving medical research. These initiatives are essential for creating robust, scalable, and interpretable AI models that can significantly advance early detection, treatment, and overall patient care in oncology. The projects leverage various AI standards to achieve these goals. Some of the key AI standards being used have been included in Table S7 in Multimedia Appendix 1. The Regulation (EU) 2018/1725, also known as GDPR, covers the fundamental requirements for access, sharing, and storage of personal data, including health data. The usage of raw health data with personal details included for research purposes remains controversial in terms of consent and audit logging. Furthermore, there is a need for standardized security and data-sharing models. Finally, conformance guidelines to ensure compliance with regulations (eg, AI Act) for manufacturers developing medical products are still needed.

## Creating Synergy Among the Involved Projects

We remarked on similarities and differences as we navigated the implementation of various standards among the projects. Despite the projects’ varied focuses, each project prioritizes interoperability, relying on various standards. This collective effort promotes knowledge exchange and innovation and fosters a digitally unified health care ecosystem for EHDS2. We also observed a trend towards common standards like HL7 FHIR and DICOM. While projects may vary in domain-specific standards and implementation nuances, leveraging established standards offers numerous advantages. This includes improved interoperability, streamlined development processes, scalability, and knowledge sharing.

Accordingly, intensive discussions accelerated the creation of the synergy among the groups that will be introduced in the Medical Informatics Europe 2024 conference [21]. In addition, both the HealthData@EU pilot and the IDERHA project were addressed as EHDS use cases in the white paper on “IoT/Edge Computing and Health Data and Data Spaces” published by the Alliance for IoT and Edge Computing Innovation [51], and both projects also shared expertise on informing EHDS2 development. Experience from completed projects working with standards helps ongoing projects to decide on which standards to focus on, learn from their limitations, etc. For example, BigPicture designed a new standard ISO/AWI 24051-2.

## Conclusion and Outlook

Standardization and legalization are the main pillars of EHDS. The HSbooster.eu initiative enabled intensive analysis of these aspects among six data-driven EU projects focusing on cancer. We aimed to create synergy among these projects to share the lessons learned in standards and legal implementation, sustainability of these projects, and harmonization with these with the EHDS2 implementation. Furthermore, we are involved in the development of standards such as ISO/IEC 21823-3:2021 “IoT - Interoperability for IoT Systems - Part 3 Semantic interoperability” (as editors), and since there is no ISO Standard for digital pathology and AI-based analysis of (whole slide) images for medical diagnosis, BigPicture is involved in ISO/PWI 24051-2 “Medical laboratories—Part 2: Digital pathology and artificial intelligence (AI)-based image analysis.”

Future work requires integration of results from TEHDAS 1&2 and HealthData@EU pilot as well as other EHDS supporting projects, such as QUANTUM [52], which is seeking to develop the EHDS data quality and utility label. Additionally, enhancements to the EHDS2 interoperability framework template are needed, with more standards covering health ontologies, wearables, personal devices, data quality, as well as the usage of FAIR principles. The template can help researchers choose appropriate standards for their projects, thereby reducing time and effort in standard selection and implementation and improving EHDS2 implementation. Moreover, the template will be used in designing and creating an interactive toolkit for the EHDS2 interoperability framework. In this way, the existing and future projects can ensure alignment with governance and interoperability requirements for EHDS2.

EHDS has recently gained additional relevance since the European AI Act that has been approved by the European Parliament by resolution on March 13, 2024, explicitly refers to the EHDS as needing to “facilitate non-discriminatory access to health data and the training of AI algorithms on those data sets, in a privacy-preserving, secure, timely, transparent and trustworthy manner, and with an appropriate institutional governance.”

## Appendix

Multimedia Appendix 1 Detailed results from analysis of six involved projects. Comparison of the usage of standards: artificial intelligence data quality.

## Abbreviations

AI: artificial intelligence
AI4HI: Artificial Intelligence for Health Imaging
ASCAPE: Artificial Intelligence Supporting Cancer Patients Across Europe
BDVA: Big Data Value Association
CDM: common data model
CMMS: Common Mandatory Metadata Structure
DCAT: Data Catalog Vocabulary
DCAT-AP: DCAT Application Profile for Data Portals in Europe
DICOM: Digital Imaging and Communications in Medicine
DQF: data quality framework
EBCP: Europe Beating Cancer Plan
EC: European Commission
EHDS: European Health Data Space
EHDS2: European Health Data Space for the secondary use of data
EIF: European Interoperability Framework
EMA: European Medicine Agency
EGA: European Genome-Phenome Archive
EU: European Union
EUCAIM: European Cancer Imaging Initiative
FAIR: Findability, Accessibility, Interoperability, and Reusability
FHIR: Fast Healthcare Interoperability Resource
GDPR: General Data Protection Regulation
HL7: Health Level 7
IDERHA: Integration of Heterogeneous Data and Evidence Towards Regulatory and Health Technology Assessments Acceptance
IEC: International Electrotechnical Commission
INHAND: International Harmonization of Nomenclature and Diagnostic Criteria
IoT: Internet of Things
ISO: International Organization for Standardization
JA: joint action
OHDSI: Observational Health Data Sciences and Informatics
OMOP: Observational Medical Outcomes Partnership
PGHD: patient-generated health data
SAREF: Smart Applications Reference Ontology
SEND: Standardization for Exchange of Nonclinical Data
SNOMED CT: Systematized Medical Nomenclature for Medicine–Clinical Terminology
TC: Technical Committee
TEHDAS: Towards the European Health Data Space
WHO: World Health Organization
WSI: whole-slide images
W3C: World Wide Web Consortium
