# Supplementary material for: Lessons Learned From European Health Data Projects With Cancer Use Cases: Implementation of Health Standards and Internet of Things Semantic Interoperability
Source: J Med Internet Res. 2025 Mar 24;27:e66273. doi: 10.2196/66273 (PMC11976176; doi:10.2196/66273)
Supplement: Multimedia Appendix 1 [file jmir_v27i1e66273_app1.docx]

**Supplementary Appendix**

**Detailed results from analysis of six involved projects.**

**Comparison of the usage of standards: AI data quality**

**Table of Contents**

Analysis of Knowledge Graphs and Related Technologies in Cancer Projects………….. Page 2

Utilizing Standards: Analyzing Health Data Spaces Projects………………………...….. Page 3

Analysis of Wearables used in the projects……………………………………………..... Page 3

Analysis of Data Quality Standards used in the projects ………………………....…….. Page 4

Analysis of Security Technologies and Standards used in the projects………….……….. Page 6

AI and Federated Standards in Cancer Research Projects in Europe………….………….. Page 7

References………….………….………………….………….…………………………… Page 9

**Tables:**

Table S1: Knowledge Graphs and Related Technologies in Cancer Projects……………... Page 2

Table S2: Standards used across six cancer-related projects……………………………..... Page 3

Table S3: Wearables used in the projects……………………………………………...…... Page 4

Table S4: Data Quality Standards used in the projects…………………….……………..... Page 5

Table S5: Security and Privacy Standards used in the projects…………………………..... Page 7

Table S6: AI/Reasoning Standards Used in the Projects……………………………..…..... Page 8

Table S7: Federated Standards Used in the Projects……………………………..……........ Page 8

**Detailed results of the conducted analysis among the six involved projects**

Analysis of Knowledge Graphs and Related Technologies in Cancer Projects

Based on the European Interoperability Framework (EIF) and principles of semantic interoperability, we compared six cancer-related projects or initiatives, looking at standards used, including health standards, interoperability standards, and standards for security and privacy.

TEHDAS guidelines, which encourage the use of ISO standards, were taken into account [1] and we highlight key standards including DCAT-AP2, OMOP, SNOMED-CT, LOINC, ISO 23494, ISO 8000-110, HL7 FHIR, and DICOM.

We conducted meetings with representatives from each of the six projects to gather the necessary information including detailing their usage of standards, and related publications.

Table S1 summarizes the utilization of knowledge graphs and related technologies, including ontologies. Ontologies compliant with HL7 FHIR standards are predominant, with additional terminologies such as SNOMED, ICD, LOINC, and OMOP being utilized in specific projects.

**Table S1: Knowledge Graphs and Related Technologies in Cancer Projects**

| Projects | Ontology Used | URL | Knowledge Graph Technologies/Terminologies |
| --- | --- | --- | --- |
| IDERHA/Lung Cancer | No HL7 FHIR ontology | Not applicable | More focused on OMOP Common Data Model, DCAT-AP: Health DCAT-AP (Planned)[2] |
| Bigpicture/Pan-Cancer whole slide images | No ontologies or Knowledge Graphs technologies | Not applicable | Terminologies such as SNOMED, ICD[3] |
| EUCAIM/Cancer Images | DICOM, DICOM Seg, OMOP, FHIR, mCODE, DCAT-AP-Health, OSIRIS | HyperOntology_v0.2 Documentation | LOINC, SNOMED, UCUM, RADLEX, ICDO3, ICD10, CPT4, ICD10PCS, ATC, NCIT, Birnlex, NAACR, Cancer [4,5] |
| iHelp/Pancreatic Cancer | FHIR TTL | FHIR TTL | Semantic Interoperability through Ontology-based Terminology Mapping: SNOMED, LOINC, ICD-9, ICD-10, UMLS Metathesaurus, OMOP-CDM, RxNorm [6-8] |
| ASCAPE/Breast and Prostate Cancer | HL7 FHIR | HL7 FHIR | Semantic Interoperability  LOINC, SNOMED CT  OMOP-CDM  ISO/CEN 13606[9,10] |
| HealthData@EU/Colorectal cancer, etc. | Not specified | Not applicable | DCAT-AP, RDF Importance of FHIR Profiles[11] |

Utilizing Standards: Analyzing Health Data Spaces Projects

Table S2 provides a comparative analysis of the standards used and highlights how each project utilizes a range of health informatics standards to ensure interoperability, security, and effective data management. Rows represent projects, and columns the standards applied.

**Table S2: Standards used across six cancer-related projects**

| Projects/ Standards | HL7 FHIR | DICOM | OMOP | ISO TC 215 | CEN TC 251 (HI Standards) | Other Standards | Knowledge Graph/ Ontology Tech |
| --- | --- | --- | --- | --- | --- | --- | --- |
| IDERHA/ Lung Cancer | Yes | Yes | Yes | Planned | - | - | DCAT-AP: HealthDCAT-AP (Planned)[2] |
| BIGPICTURE/Pan-Cancer whole slide images | - | Yes | - | Yes | - | SNOMED, ICD | - |
| EUCAIM/Cancer Images | - | Yes | Yes | Yes | - | - | - |
| iHelp/ Pancreatic Cancer | Yes | - | Yes | - | Yes | SNOMED LOINC, ICD-9, ICD-10, UMLS Metathesaurus, SPARQL, RDFS, RxNorm | - |
| ASCAPE/ Breast& Prostate Cancer | Yes | - | - | Yes | Yes | LOINC, SNOMED | - |
| HealthData@EU/Colorectal Cancer | - | - | - | - | Not in the cancer genomics use case | - | DCAT-AP: HealthDCAT-AP |

Analysis of Wearables used in the projects

Advancements in IoT, mobile applications, and wearables have unlocked new potentials within the digital healthcare domain. These devices serve two primary functions: firstly collecting lifestyle and behavioral data and secondly facilitating direct communication between healthcare professionals and individuals. This direct communication includes delivering AI-based personalized early risk identification and decision support through notifications, messages, and personalized recommendations.[12]

Data from sensors, mobile, and wearable devices are integrated with clinical data from medical experts to enhance insights by processing and analyzing all health determinants of individuals[6]. For example, the iHelp project employs HL7 FHIR standard-compatible data structures to integrate primary and secondary data, compiling them into the Holistic Health Records (HHRs) models[6]. HL7 FHIR is suitable for clinical and streaming data from sensors.[8] Table S3 gives a summary of wearables used in the projects.

**Table S3: Wearables used in the projects**

| Projects | Related Condition | Wearables/Sensors/Devices | Patient-Generated Health Data(PGHD) |
| --- | --- | --- | --- |
| IDERHA | Lung Cancer | Withings Scan Watch 2 (Planned) | Heart rate, blood oxygen, steps per day |
| Bigpicture | Pan-Cancer whole slide images | - | Pathology images |
| EUCAIM | Cancer Images | - | Cancer images |
| iHelp | Pancreatic Cancer | Garmin, Fitbit, Healthentia mobile application[6,8,13] | Steps, distance, elevation, energy, heart rate, sleep stages, as well as questionnaires on diet, sleep quality/quantity, smoking, pain, nutrition |
| ASCAPE | Breast and Prostate Cancer | FitBit, various mobile devices[10] | Steps, activity type/time, calories burned, heart rate, sleep quality/quantity |
| HealthData@EU | Colorectal Cancer, etc. | - | - |

Analysis of Data Quality Standards used in the projects

Ensuring the accuracy, completeness, consistency, and reliability of cancer research data is of utmost importance, and adherence to data quality standards plays a crucial role in achieving this goal. These standards not only help maintain data integrity but also promote data interoperability through the use of standardized data models and vocabulary. This enables seamless data exchange and integration across different projects and platforms. Additionally, adherence to these standards supports informed decision-making by providing high-quality data for clinical decisions, research insights, and cancer treatment and prevention policymaking. Compliance with international legal and regulatory requirements is also ensured, upholding proper data governance and ethical data usage. Lastly, establishing a unified framework for data quality encourages collaboration among various stakeholders within the cancer research community.

Table S4 presents an analytical view of the Data Quality Standards used in the projects.

**Key Quality Standards Used in at least one project:**

1. ISO/IEC FDIS 5259 Series: Focuses on data quality for AI systems, particularly for analytics and ML, providing comprehensive guidelines and frameworks to ensure the quality and reliability of data used in AI applications.
2. ISO 8000 Series: Defines principles and guidelines for exchanging, integrating, sharing, and maintaining accurate, complete, and consistent data, crucial for decision-making and operational efficiency.
3. ISO 9000: Provides guidelines for establishing, implementing, maintaining, and improving an organization's quality management system (QMS), focusing on enhancing customer satisfaction.
4. ISO 13485: Specifies QMS requirements for organizations involved in the design, development, production, installation, and servicing of medical devices, ensuring regulatory compliance and safety.
5. ISO/IEC 25012: Defines a set of software quality metrics related to data quality, focusing on characteristics such as accuracy, completeness, consistency, and data reliability.
6. ISO/IEC 25010: Specifies a model for evaluating software product quality, outlining characteristics such as functionality, reliability, usability, efficiency, maintainability, portability, compatibility, and security.
7. ISO/IEC 25040: Provides guidelines for evaluating software product quality, detailing a systematic approach for consistent and comprehensive assessments.
8. ISO/IEC 25023: Specifies measures for assessing software product quality characteristics, including performance efficiency, compatibility, usability, reliability, security, maintainability, and portability.
9. ISO/IEC 25024:2015: Focuses on data quality measures, providing a framework to assess data quality used in software systems, ensuring data is accurate, complete, and fit for its intended use.
10. ISO 13972:2022: Establishes requirements for clinical information models used in healthcare, ensuring clinical data is accurate, consistent, and reusable, supporting decision-making and interoperability.
11. ISO 14971 provides a comprehensive framework for risk management in medical device manufacturing, emphasizing identifying, evaluating, and controlling risks to ensure safe devices. This standard enhances medical devices' reliability, safety, and regulatory compliance.
12. DCAT-AP: A specification for describing European public sector datasets, enabling cross-data portal search and improving public sector data accessibility and searchability.
13. W3C Standards for Semantic Web: Including RDF, RDFS, OWL, and SPARQL, these standards form the backbone of semantic web infrastructure, facilitating interconnected data and knowledge discovery

**Table S4: Comparison of Data Quality Standards used in the projects**

| Standards/ Projects | IDERHA | Bigpicture | EUCAIM | iHelp | ASCAPE | HealthData@EU |
| --- | --- | --- | --- | --- | --- | --- |
| ISO/IEC FDIS 5259 Series (AI) | No | No | No | Yes | No | No |
| ISO/IEC 25012:2008 | No | No | Yes | Yes | No | No |
| ISO 8000 Series | No | No | No | No | No | No |
| ISO 9000 Series | No | No | Yes | Yes | No | Yes |
| ISO 13485 | Yes | No | No | No | No | No |
| ISO/IEC 25010 | No | No | Yes | Yes | No | No |
| ISO/IEC 25040 | No | No | Yes | Yes | No | No |
| ISO/IEC 25023 | No | No | Yes | Νο | No | No |
| ISO/IEC 25059:2023 | No | No | No | No | No | No |
| ISO 14971 | Yes | No | No | No | No | No |
| W3C DCAT-AP | No | No | Yes | No | Yes | Yes |
| W3C Data Quality Vocabulary (DQV) | Planned | No | Yes | No | Yes | Yes |
| ISO/IEC 25024:2015 | No | No | Yes | Yes | No | No |
| ISO 13972:2022 | No | No | No | No | No | No |

**Analysis of Security Technologies and Standards used in the projects:**

Security is a paramount concern in healthcare and cancer research projects, necessitating adherence to rigorous standards and regulations to safeguard sensitive data and ensure user privacy. The following standards and regulations related to security are commonly employed:

1. ISO/IEC 27001: Recognized as an international benchmark for information security management systems (ISMS), ISO/IEC 27001 provides a comprehensive framework for identifying, assessing, and mitigating information security risks. In healthcare projects, compliance with ISO/IEC 27001 ensures the implementation of robust security controls to protect patient data from unauthorized access, disclosure, or tampering.
2. ISO/IEC 27002: Complementary to ISO/IEC 27001, ISO/IEC 27002 offers detailed guidelines and best practices for implementing specific security controls within an ISMS. It covers various aspects of information security, including organizational security, access control, cryptography, and incident management. Adhering to ISO/IEC 27002 helps healthcare organizations establish a layered defense strategy to counter evolving cybersecurity threats effectively.
3. ISO 27799:2016: Tailored specifically for the healthcare sector, ISO 27799 provides security guidelines for managing health information within healthcare organizations. It addresses the unique challenges and regulatory requirements of protecting sensitive patient data, such as medical records and diagnostic information. By adhering to ISO 27799, healthcare providers can establish robust security measures to ensure the confidentiality, integrity, and availability of health information systems.
4. HL7 Healthcare Privacy and Security Classification System (HCS): Developed by HL7, the HCS framework enables healthcare organizations to classify and manage healthcare information based on privacy and security requirements. It provides a standardized approach to assessing and mitigating privacy and security risks associated with exchanging healthcare data. Integration of the HCS framework ensures that sensitive patient information is handled by regulatory standards and industry best practices.
5. SMART on FHIR (extending OAuth2.0): An open, standards-based platform that extends OAuth 2.0 to enable secure authorization for FHIR-based applications. It provides a framework for building healthcare applications that can integrate seamlessly with Electronic Health Records (EHRs), patient portals, and other health IT systems. SMART on FHIR leverages OAuth 2.0 to ensure that applications can obtain access tokens to interact with FHIR resources securely, supporting scopes that specify fine-grained access control to health data.
6. ISO/IEC 42001-2023: As the world's inaugural standard on AI management systems, ISO/IEC 42001-2023 is pivotal in ensuring artificial intelligence's security and ethical use in healthcare settings. By providing guidelines for managing AI systems, including risk assessment, governance, and compliance, ISO/IEC 42001-2023 helps healthcare organizations mitigate the security risks associated with AI deployment and promote responsible AI innovation.
7. ISO/TS 22600: Guidelines for privilege management and access control in health information systems.
8. ISO/TS 21298, "Health Informatics – Functional and Structural Roles," establishes a standard framework in health informatics. It delineates guidelines for defining functional and structural roles within health information systems, fostering clarity and consistency in role delineation across diverse healthcare environments.
9. The General Data Protection Regulation (GDPR) is an EU law designed to enhance data protection for individuals. It applies to any organization handling the personal data of EU residents, irrespective of the organization's location. Key components of GDPR include Data Protection by Design and Default, Security of Processing, Data Breach Notification, Rights of Data Subjects, and Accountability and Governance. GDPR ensures robust data protection and privacy practices, enhancing individuals' control over their personal data.

By incorporating these standards and regulations into healthcare and cancer research projects, organizations can establish a robust security posture, mitigate cybersecurity risks, and safeguard the confidentiality, integrity, and availability of sensitive data. For example, IDERHA ensures the evaluation of personal data with privacy-preserving and distributed analytics while achieving “federated data resources ” (i.e., stored, managed, and controlled by the data providers at their facilities). “Authentication and authorization services to manage secure data access” is planned within IDERHA.[2] Security and Privacy Ontology based on HL7 and ISO is designed within the ACTIVAGE EU project and association as well [14,15]. HL7 Security and Privacy Ontology comes from HL7 Healthcare Privacy and Security Classification System (HCS), ISO/TS 21298 Health Informatics – Functional and structure roles, and ISO/TS 22600 Health Informatics- Privilege management and access control 2006.[16] In addition, EUCAIM uses Life Sciences Authentication and Authorisation Infrastructure (LS-AAI).[17] This is written in several deliverables, for example, EUCAIM D4.5.[18] with industry standards fosters trust among stakeholders and demonstrates a commitment to upholding the highest standards of security and privacy in healthcare practices.[11,13] The GDPR is utilized in all projects and is a fundamental aspect of security in Europe.[19]

Table S5 provides a comparative overview of the security standards applied in different healthcare and cancer research projects, highlighting their focus on ensuring data confidentiality, integrity, and availability while complying with industry regulations and best practices:

**Table S5: Security and Privacy Standards used in the projects**

| Standards/Projects | IDERHA | Bigpicture | EUCAIM | iHELP | ASCAPE | HealthData@EU |
| --- | --- | --- | --- | --- | --- | --- |
| ISO/IEC 27001 | Yes | Yes[20] | Yes | Yes[6] | - | - |
| ISO/IEC 27002 | - | - | - | - | - | - |
| ISO 27799:2016 | - | - | - | Yes | - | - |
| HL7 HCS | - | - | - | Yes | - | - |
| SMART on FHIR (extending OAuth2.0) | - | - | - | Yes | - | - |
| ISO/IEC 42001-2023 | - | - | - | - | - | - |
| ISO/TS 21298 | Yes | - | - | - | - | - |
| ISO/TS 22600 | Yes | - | - | - | - | - |
| GDPR | Yes | Yes | Yes | Yes | Yes[21] | Yes |

AI and Federated Standards in Cancer Research Projects in Europe

Integrating and analyzing information from diverse sources presents a significant challenge in cancer research and is crucial for various stakeholders. Artificial Intelligence (AI) and effective big data management play pivotal roles in enabling tailored data-driven approaches aimed at early risk prediction, prevention, and intervention.[6] In Europe, AI and federated learning are essential components in advancing personalized cancer care. Projects such as IDERHA, EUCAIM, and ASCAPE utilize federated data infrastructures and federated learning strategies to ensure privacy and foster collaboration without centralized data storage. Key standards like ISO/AWI 24051-2 and OMOP-CDM are fundamental in standardizing and harmonizing data, facilitating efficient federated analytics.

The adoption of federated standards and AI in cancer research projects across Europe represents a shift towards collaborative yet privacy-preserving medical research. These initiatives are essential for creating robust, scalable, and interpretable AI models that can significantly advance early detection, treatment, and overall patient care in oncology.[22] The projects leverage various AI standards to achieve these goals. Some of the key AI standards being used include:

1. ISO/IEC 42001_2023 – Artificial Intelligence – Management System, which marks the world’s inaugural standard on AI management systems. This pioneering standard establishes a precedent for incorporating artificial intelligence into healthcare frameworks, guaranteeing resilient management procedures and ethical deliberations in AI deployment.
2. ISO/AWI 24051-2 Medical laboratories — Part 2: Digital pathology and artificial intelligence (AI)-based image analysis. This upcoming standard aims to specify requirements and to give recommendations for the digitalization of slide-mounted, stained sections, processing of digital whole slide images, and artificial intelligence (AI) based image analysis.
3. ISO/IEC 22989 - Information technology — Artificial intelligence — Artificial intelligence concepts and terminology: It is instrumental in guiding the development, deployment, and management of AI systems by also defining what constitutes a "machine learning model" or "neural network" within the project's documentation and discussions.
4. ISO/IEC JTC 1/SC 42 Artificial Intelligence: This subcommittee addresses standardization in the area of Artificial Intelligence. Its scope includes foundational standards, AI computational approaches, AI trustworthiness, and ethical and societal concerns. The work of ISO/IEC JTC 1/SC 42 is crucial for ensuring that AI technologies are developed and deployed in a reliable, safe, and ethically sound manner.

**Table S6: AI/Reasoning Standards Used in the Projects**

| Projects | Standards | Techniques |
| --- | --- | --- |
| IDERHA/ Lung Cancer | Federated data infrastructure | Federated Machine Learning (FML) algorithms and federated data resources managed and controlled by data providers at their facilities.[2] |
| BigPicture/ Pan-Cancer whole slide images | ISO/IEC 42001:2023, ISO/AWI 24051-2 (under development), ISO/IEC JTC 1/SC 42 | AI-based image analysis.[3] |
| EUCAIM/ Pan-Cancer Images | OMOP-CDM for standardization and harmonization | The federated infrastructure of cancer-related images, enabling distributed network research and federated analytics.[2,23] |
| iHelp/ Pancreatic Cancer | ISO/IEC 22989, OMOP-CDM for standardization and harmonization | Advanced DevOps and ML tools (ML Flow, Jupyter Notebooks, Docker, Kubernetes)[24], frugal AI algorithms, predictive algorithms (LightGBM, SVM, XGBoost, Random Forest),[6,7] Deep Learning Models, Explainable AI (XAI), automated deployment using Kubernetes.[25] |
| ASCAPE/ Breast and Prostate | ISO/IEC 42001:2023 | XAI techniques, federated deep learning, privacy-preserving data processing (homomorphic encryption)[26], AI models for personalized cancer care, and open AI infrastructure.[27,28] |
| HealthData@EU/ Colorectal Cancer |  | No direct application of AI in cancer genomics. Federated querying only.[29] |

Federated approaches are crucial for leveraging distributed datasets while safeguarding patient privacy and data sovereignty. Most projects employ federated strategies custom-made to specific objectives, and harnessing technologies like Federated Machine Learning (FML) and federated data infrastructures.

**Table S7: Federated Standards Used in the Projects**

| Projects | Federated Learning | Description |
| --- | --- | --- |
| IDERHA | Yes | Utilizes federated data infrastructure to manage and control data at provider facilities[2] |
| BigPicture | No | Data is currently collected in a central repository (mirrored in two locations); POC for federated EGA technology[3] |
| EUCAIM | Yes | OMOP-CDM standardizes healthcare information for distributed research and federated analytics[23] |
| iHelp | Yes | Utilizes federated queries to access and analyze distributed data sources, facilitating personalized health monitoring [6,8] |
| ASCAPE | Yes | Implements incremental and semi-concurrent federated learning schemes for personalized cancer-care predictive models[26,27,30] |
| HealthData@EU | Yes | Uses federated queries for distributed data analysis[29] |

References:

1. TEHDAS project. EU-wide collaboration needed to optimise health data use for research and innovation [Internet]. Tehdas. 2023 [cited 2024 Jul 4]. Available from: <https://tehdas.eu/tehdas1/results/eu-wide-collaboration-needed-to-optimise-health-data-use-for-research-and-innovation/results/eu-wide-collaboration-needed-to-optimise-health-data-use-for-research-and-innovation/>

2. Hussein R, Balaur I, Burmann A, Ćwiek-Kupczyńska H, Gadiya Y, Ghosh S, et al. Getting ready for the European Health Data Space (EHDS): IDERHA’s plan to align with the latest EHDS requirements for the secondary use of health data. Open Research Europe In press. 2024;

3. BigPicture Report D5.05. Landscape of Guidelines, Standards and Regulatory Requirements Relevant For Digital Pathology (Clinical) [Internet]. 2023 Apr [cited 2024 Jul 2]. (WP5 - Regulatory framework for digital slides and AI-based methods). Report No.: 945358. Available from: <https://bigpicture.eu/deliverables-reports>

4. Kalokyri V, Kondylakis H, Sfakianakis S, Nikiforaki K, Karatzanis I, Mazzetti S, et al. MI-Common Data Model: Extending Observational Medical Outcomes Partnership-Common Data Model (OMOP-CDM) for Registering Medical Imaging Metadata and Subsequent Curation Processes. JCO Clin Cancer Inform. 2023 Sep;(7):e2300101.

5. LIMICS. EUCAIM’s HyperOntology_v0.2beta. 2024 May 3 [cited 2024 Jul 2]; Available from: <https://zenodo.org/records/11109765>

6. G. Manias, H. Op Den Akker, A. Azqueta, D. Burgos, N. D. Capocchiano, B. L. Crespo, et al. iHELP: Personalised Health Monitoring and Decision Support Based on Artificial Intelligence and Holistic Health Records. In: 2021 IEEE Symposium on Computers and Communications (ISCC). 2021. p. 1–8.

7. Manias G, Kouremenou E, Alzúaz AA, Kranas P, Melillo F, Kyriazis D. An Optimized Pipeline for the Processing of Healthcare Data towards the Creation of Holistic Health Records. In: 2023 International Conference on Applied Mathematics & Computer Science (ICAMCS) [Internet]. 2023 [cited 2024 Jul 2]. p. 50–6. Available from: https://ieeexplore.ieee.org/document/10438686

8. Manias G, Azqueta-Alzúaz A, Dalianis A, Griffiths J, Kalogerini M, Kostopoulou K, et al. Advanced Data Processing of Pancreatic Cancer Data Integrating Ontologies and Machine Learning Techniques to Create Holistic Health Records. Sensors. 2024 Jan;24(6):1739.

9. Frid S, Fuentes Expósito MA, Grau-Corral I, Amat-Fernandez C, Muñoz Mateu M, Pastor Duran X, et al. Successful Integration of EN/ISO 13606–Standardized Extracts From a Patient Mobile App Into an Electronic Health Record: Description of a Methodology. JMIR Med Inform. 2022 Oct 12;10(10):e40344.

10. Fuentes A, Amat C, Lozano-Rubí R, Frid S, Muñoz M, Escarrabill J, et al. mHealth Technology as a Help Tool during Breast Cancer Treatment: A Content Focus Group. Int J Environ Res Public Health. 2023 Mar 4;20(5):4584.

11. HealthData@EU Pilot: Milestone M6.1. Report on the landscape analysis of available metadata catalogues and the metadata standards in use [Internet]. 2023 Mar [cited 2024 Feb 7]. Available from: https://ehds2pilot.eu/wp-content/uploads/2024/04/HealthData@EU-Pilot_MS6.1_FIN.pdf

12. Huang Y, Upadhyay U, Dhar E, Kuo LJ, Syed-Abdul S. A Scoping Review to Assess Adherence to and Clinical Outcomes of Wearable Devices in the Cancer Population. Cancers. 2022 Jan;14(18):4437.

13. Pnevmatikakis A, Kanavos S, Matikas G, Kostopoulou K, Cesario A, Kyriazakos S. Risk Assessment for Personalized Health Insurance Based on Real-World Data. Risks. 2021 Mar;9(3):46.

14. activage association | activage association [Internet]. 2023 [cited 2024 Jul 3]. Available from: <https://activage-association.org/>

15. ACTIVAGE Project : Internet of Things (IoT) for ageing well [Internet]. Activage Project. [cited 2024 Jul 3]. Available from: http://www.activageproject.eu/index.html

16. DevTools-SILtools-SemanticsEditor/docroot/resources/ontologies/SecurityAndPrivacyOntology.owl at master · AIoTES/DevTools-SILtools-SemanticsEditor [Internet]. GitHub. [cited 2024 Jul 3]. Available from: <https://github.com/AIoTES/DevTools-SILtools-SemanticsEditor/blob/master/docroot/resources/ontologies/SecurityAndPrivacyOntology.owl>

17. EIBIR.org [Internet]. [cited 2024 Jul 3]. Available from: <https://www.eibir.org>

18. Achievements [Internet]. Cancer Image Europe. [cited 2024 Jul 3]. Available from: <https://cancerimage.eu/achievements/>

19. DPM. What is the Difference Between GDPR and ISO 27001 [Internet]. Data Privacy Manager. 2021 [cited 2024 Jul 4]. Available from: <https://dataprivacymanager.net/what-is-the-difference-between-gdpr-and-iso-27001/>

20. Deliverables & Reports | Bigpicture [Internet]. [cited 2024 Jul 3]. Available from: <https://bigpicture.eu/deliverables-reports>

21. Tzelves L, Manolitsis I, Varkarakis I, Ivanovic M, Kokkonidis M, Useros CS, et al. Artificial intelligence supporting cancer patients across Europe—The ASCAPE project. PLOS ONE. 2022 Apr 21;17(4):e0265127.

22. Boutros M, Baumann M, Bigas A, Chaabane L, Guérin J, Habermann JK, et al. UNCAN.eu: Toward a European Federated Cancer Research Data Hub. Cancer Discovery. 2024 Jan 12;14(1):30–5.

23. Kondylakis H, Kalokyri V, Sfakianakis S, Marias K, Tsiknakis M, Jimenez-Pastor A, et al. Data infrastructures for AI in medical imaging: a report on the experiences of five EU projects. Eur Radiol Exp. 2023 May 8;7(1):20.

24. Danciu G, Nicolae IE, Ilie I, Nechifor CS. Advanced Notebook: A tool for enhanced Management of Machine Learning models and procedures in the Healthcare Domain. In: 2023 International Conference on Applied Mathematics & Computer Science (ICAMCS) [Internet]. 2023 [cited 2024 Jul 4]. p. 36–41. Available from: <https://ieeexplore.ieee.org/document/10438681>

25. Kranas P, Kolev B, Levchenko O, Pacitti E, Valduriez P, Jiménez-Peris R, et al. Parallel query processing in a polystore. Distrib Parallel Databases. 2021 Dec 1;39(4):939–77.

26. Popescu AB, Taca IA, Vizitiu A, Nita CI, Suciu C, Itu LM, et al. Obfuscation Algorithm for Privacy-Preserving Deep Learning-Based Medical Image Analysis. Applied Sciences. 2022;12(8).

27. Savić M, Kurbalija V, Ilić M, Ivanović M, Jakovetić D, Valachis A, et al. The application of machine learning techniques in prediction of quality of life features for cancer patients. Computer Science and Information Systems. 2023;20(1):381–404.

28. J. Rust, S. Autexier. Causal Inference for Personalized Treatment Effect Estimation for given Machine Learning Models. In: 2022 21st IEEE International Conference on Machine Learning and Applications (ICMLA). 2022. p. 1289–95.

29. Data interoperability, quality and protection - EHDS2 Pilot - Official website [Internet]. 2022 [cited 2024 Jul 4]. Available from: https://ehds2pilot.eu/package/citizens/

30. Popescu AB, Taca IA, Nita CI, Vizitiu A, Demeter R, Suciu C, et al. Privacy Preserving Classification of EEG Data Using Machine Learning and Homomorphic Encryption. Applied Sciences. 2021;11(16).
